# Relationships and boundaries: Learning needs and preferences in clerkship medical environments

**DOI:** 10.1371/journal.pone.0236145

**Published:** 2020-07-20

**Authors:** Tahra AlMahmoud, M. Jawad Hashim, Naghma Naeem, Rabah Almahmoud, Frank Branicki, Margaret Elzubeir

**Affiliations:** 1 Department of Surgery, College of Medicine and Health Sciences, United Arab Emirates University, Al Ain, UAE; 2 Department of Family Medicine, College of Medicine and Health Sciences, United Arab Emirates University, Al Ain, UAE; 3 Department of Medical Education, College of Medicine and Health Sciences, United Arab Emirates University, Al Ain, UAE; 4 Department of Clinical Sciences, College of Medicine, University of Sharjah, Sharjah, UAE; Technion - Israel Institute of Technology, ISRAEL

## Abstract

**Purpose:**

Relationship boundaries recognition is an essential element of medical practice. The aim of the study was to assess final year medical students’ perceived need for education regarding professional boundaries.

**Materials and methods:**

This was a cross-sectional study. An anonymous paper questionnaire was distributed to 128 final year medical students. Standard descriptive statistics, unpaired t-test to evaluate differences between male and female groups and Pearson correlation to determine relationships between variables were used.

**Results:**

The survey was completed by 84.4% of students who identified the need for more emphasis in the curriculum for all of topics during training and practice pertaining to boundaries and relationships (mean 6.61±1.32 on a scale of 0 to 9; and 6.66±1.27 respectively). Topics with a high interest ranking requiring additional attention were mistreatment of medical students (mean 7.22±1.96), coping with mistakes in clinical care (mean 7.25±1.63), reporting of medical mistakes (mean 7.58±1.36), and gender bias in clinical care (mean 7.10±1.82). Women perceived a greater need for attention to all topics in the curriculum. Significant differences between the perceptions of female and male students were observed regarding topics such as responding to an impaired colleague (p<0.001), and a physician’s social responsibilities (p = 0.001).

**Conclusion:**

Medical students recognized the need for more education and training in the undergraduate medical ethics curriculum regarding patient-physician relationship boundaries.

## Introduction

Boundaries perception and appreciation of cultural norms are merits that guides acceptable professional conduct in medical practice. [1, 2] It has been shown that the main reasons for physician disciplinary actions includes needless prescription, violations of boundaries with patients, and fraud. [3] It has also been evident that deficiency in professionalism among medical students are early signals for unsafe clinical performance. [4] In contemporary medical practice; patient care is a shared responsibility between patients, physicians (who are often from different medical specialties), and allied health professionals; professional boundaries are complex. While adherence to ethical principles and behaviors remains fundamental aspects of professionalism, [5] it is essential that medical trainees become familiar with physician-patient relationship boundaries. [6] Noticeably, ethics curricula generally target more striking concerns such as abortion and euthanasia rather than more subtle issues. [7] Ethical scenarios such as medical students’ being introduced to patients as “doctors”, or being asked to write prescriptions for co-workers that may occur in daily practice merit greater awareness amongst students in a formal curriculum and day-to-day clinical practice. Based on available evidence featuring medical students’ recognition of greater needs for ethical and professional training [8–12] along with concerns for not being able to maintain some of their doctor-patient relationship scopes. [13] We hypothesized that medical students in our context will sense the need for increased curricular consideration of topics pertaining to relationships and boundaries.

In this study, we assessed the perceptions of clinical medical students in final years (in ‘clerkships’) regarding relationship boundaries, an area that has received little attention in medical education research. We also explored the differences between subgroups and examined correlations that may explain students’ views about educational need for issues pertaining to relationship boundaries. This study was conducted based on expectation that an understanding of medical students’ experiences and perspectives with regard to professional boundaries may help promote favorable medical practice.

## Methods

### Theoretical perspectives

#### Definitions

Prior to discussion about clinical practice, it is pertinent to define professional boundaries as rules that establish a professional relationship. [14] Boundaries are necessary for the growth, functioning and maintenance of relationships and guard against objectionable relationships. [15] According to Gutheil and Gabbard [16] boundaries can be defined and crossed regarding role, time, place and space, money, gifts, clothing, language, self-disclosure, physical contact and use of technology in clinical practice.

Several terms have been used to describe dysfunctional professional relationships within clinical practice including boundary transgression, boundary crossing and boundary violations. Boundary transgressions range from crossings to violations and are all associated with breaches in professional conduct and deviations from accepted practice. A violation is at the extreme end of the spectrum and has been described as a harmful crossing of a boundary. [16] Professionals are guided and regulated in their clinical practice by professional codes of ethics. [6, 17, 18]

#### Ethics

Ethical theories that apply to all health care professionals are Deontological, Utilitarian and Virtue. [19] Deontological ethics relates to a professional’s moral obligation and commitment to beneficence and non-malfeasance. Utilitarian ethics posits that there should be maximum benefit for as many patients as possible and that all patients are treated equally. Virtue ethics is most relevant in the context of clinical practice as it is concerned with justice, compassion, and fidelity. Fidelity, in particular, deals with a caregiver held in trust to behave appropriately, and is important in establishment and maintenance of professional relationships and boundaries. Thus, fidelity forms the basis on which professional relationships are established and how professional boundaries are demarcated.

#### Boundaries

The concept of boundaries dates back to the Hippocratic Oath, which sets down some parameters for physicians: “…all intentional ill-doing and all seduction, and especially from the pleasures of love with women and men.”

Sigmund Freud’s Psychoanalytic Theory recognized a defining line in the relationship between the physician and the patient [20] forming the basis of later discourse about the need for boundaries. Professional boundaries have also been described in Psychodynamics and Psychotherapy Theories. The concept of boundaries in mental health clinical practice was first presented in a seminal report by Gutheil and Gabbard. [16] Later, Sule [21] described three functional spaces that are created when boundaries are used professionally. These spaces are the reflective process in clinical practice, internal spaces of both the patient and the clinician and the interactive space between them. According to Peterson [22] boundaries are violated when professionals abuse their power and privilege to transgress into the space which exists between the patient and doctor, thereby jeopardizing patient safety.

#### Professional relationships

Development and Relational Theory is pertinent to the formation of professional relationships as described by Hill and Knox. [23] Relational theory proposes that a central human necessity is the establishment of an authentic and mutual connection in the relationship. Our needs for touch, love, interaction, and responsiveness exist from the time of birth [24] life experiences playing an important role in future relational expectations [25]. Developmental and relational theories also hypothesize that self is co-constructed by interaction between relational experiences and interactions, and is also determined by social and cultural influences. [26] Therefore, early development experiences and relationships, later interactions, the culture and society to which individuals belong, all affect expectations in the doctor-patient relationship.

### Instrument

A paper-based survey was distributed to final year medical students with a front letter expressing the purpose of the questionnaire and anonymity procedures. Participants had the choice to complete the survey or decline as implied consent which is standard practice for non-clinical questionnaires. Ethical approval for the study was granted by Al Ain Medical District Human Research Ethics Committee- Protocol No.10/40. The survey was developed at the University of New Mexico, USA (copyright reserved by Laura Roberts, Cynthia Geppert and Teddy Warner; permission to use the instrument was obtained). The original survey covered multiple domains with regards to professionalism and ethics education. [11, 27, 28] All components focus on ethically important dilemmas physicians in training are likely to encounter in their day to day activities. We postulated that the academic medicine community would benefit from in-depth exploration of individual domains in dedicated publications; thus reducing potential for significance or subtleties of domains being overlooked. One domain has detailed question items on attitudes, goals, learning methods, curricula, knowledge assessment, and skills assessment. [29] Another domain has directed question items related to educational needs concerning informed consent topics, principles, and vulnerable populations. [30] The last domain concentrates on question items linked to relationship and boundaries. This manuscript focuses on medical students’ perception for educational needs compared with current curricular exposure regarding the relationship and boundaries domain. Each item was rated on a 9-point scale addressing the level of educational attention needed compared with that currently provided. The items were scaled from 1 (i.e., need much less attention) to 9 (i.e., need much more attention). The instrument has been shown to have good reliability and has been used internationally. [5, 27, 28]

## Research context and participants

This descriptive, cross-sectional study was conducted at the College of the Medicine & Health Sciences (CMHS) United Arab Emirates (UAE) University. The CMHS offers a six-year MD undergraduate program consisting of two years each of premedical and preclinical phases followed by two years of clinical clerkships. The CMHS curriculum is outcomes/competency based and learning strategies include lectures, small group, and interactive learning opportunities such as Team-based and Problem-based learning. During clinical clerkships students rotate in core clinical specialty attachments for periods of between four and eight weeks. The six Accrediting Council for Graduate Medical Education [ACGME 1999] outcomes were adopted in our MD program. These include medical knowledge, patient care and procedural skills, professionalism, interpersonal and communication skills, practice based learning and improvement and systems based practice. As is the case for all outcomes, Professionalism is taught with increasing theoretical and practical application from Year 1 to year 6 in our spiral curriculum. In clerkships the professionalism core component speaks directly to the attitudes and behaviors expected of students on graduation from the MD program. To successfully master this attribute students must demonstrate adherence to ethical principles and commitment to carrying out professional responsibilities. Respect, altruism, honesty, integrity, compassion and empathy are taught, role modelled and expected in all interactions with patients and their families as well as with peers, supervisors and members of the multidisciplinary team. Demonstration of professional conduct and accountability is therefore expected throughout the six-year program and there are opportunities for relationship boundary issues to be addressed. Similarly, the unique aspects of culture, race, gender, age and religion are incorporated into the teaching of the bio-psycho-social model of health and well-being. Students understand that they are accountable not only to patients but also to peers/colleagues and society in general.

Research subjects were final year medical students at the College of Medicine and Health Sciences, United Arab Emirates (UAE) University. The UAE University is the largest and oldest university in the country. As a federally funded institution, it is mandated to provide higher education to UAE citizens. Approximately 100 medical students are admitted each year to the six-year MD program, which includes clinical training in the final two years. The vast majority of students are Emirati nationals of Arab ethnic and cultural background.

The survey was administered to all students during the surgical specialty clerkship, August 2009 to February 2013. This clerkship is mandatory for all final year medical students and is distinct from the general surgery clerkship. Participants had formerly finished clerkships in Internal Medicine, Surgery, Family Medicine and Psychiatry. The final year students were included in the study as the aim was to probe the opinion of participants who are familiar with the entire curriculum and are anticipated to have developed and experienced some professional attitudes and behaviors. The annual number of student intake has been small until recently, hence, the questionnaire was distributed over several academic years for sample size purposes. All students were exposed to the same formal curriculum and the potential for learning opportunities and clinical experience are similar for male and female.

## Data analysis

Descriptive statistics of each item in the survey were attained. Cronbach’s α, the unpaired *t* test and correlation coefficients (r) were assessed. All analyses were carried out using SPSS for Windows version 20.0 (SPSS, Chicago, ILL). A *P* value <0.05 was considered significant. Items were grouped as either training or practical topics based on conceptual coherence.

## Results

A total of 128 surveys (32 males, 96 females) were distributed among final year medical students, with an 84.4% response rate (24 males and 84 females). Mean age of participants was 24 years and the majority being unmarried (85%).

### Educational needs concerning ethically important practices and behaviors related to relationships and boundaries during the training period (academic-educational)

Final year students indicated the need for more attention to all relationship and boundary topics during their training (mean 6.61±1.32). Topics for which more additional attention was requested were mistreatment of medical students (mean7.22±1.96), coping with mistakes in clinical care (mean7.25±1.63), and reporting of medical mistakes (mean7.58±1.36) (Table 1). Female students statistically significantly requested more attention to the following topics when compared with male students: performing work beyond one’s competence (p = 0.004), resolving conflicts between allied health professionals (p = 0.000), personal relationships with patients (0.000), mistreatment of medical students (p = 0.009), adequately caring for patients while adhering to work-hour guidelines (p = 0.001), and being asked to work with inadequate supervision (p = 0.000) (Table 1). Most significant differences were observed between males and females concerning the following items: responding to an impaired (e.g. drug or alcohol abuse) colleague (mean7.31±1.46 versus 5.88±1.51, p = 0.000), and the physician’s social and political responsibilities (7.04±1.75 versus 5.13±2.22, p = 0.001) (Table 1).

**Table 1:** Educational needs as to ethically important practices and behaviors related to relationships and boundaries during the training period (academic-educational) (Mean±SD).

	Gender		Overall (N = 108)
Behaviors (α0.880)	Female (N = 84)	Male(N = 24)	Male and female
1. Learning procedures on cadavers	6.65±2.09*	5.21±2.47	6.33±2.25
2. Confidentiality of medical records	6.87±1.80*	6.00±1.72	6.68±1.81
3. Performing work beyond one’s competence	6.66±1.81*	5.50±1.29	6.40±1.77
4. Resolving conflicts between attending physician and students	7.17±1.51	6.50±1.53	7.02±1.53
5. Mistreatment of medical students	7.54±1.74*	6.13±2.31	7.22±1.96
6. Mistreatment of interns and residents	7.18±1.72*	5.58±2.34	6.82±1.98
7. Student health care	7.37±1.61*	6.17±2.22	7.10±1.82
8. Students introduced to patients as “doctors”	6.69±1.96	6.13±2.56	6.56±2.11
9. Adequately caring for patients while adhering to work-hour guidelines	6.95±1.61*	5.67±1.95	6.66±1.76
10. Being asked to work with inadequate supervision	6.94±1.74*	5.29±1.90	6.57±1.89
11. Sexual/romantic relationships between faculty and medical students	6.05±2.37	5.67±2.73	5.96±2.45
12. Sexual/romantic relationships between interns, residents and medical students	5.89±2.43	5.71±2.79	5.85±2.50
**Group means**	6.84±1.28*	**5.80±1.11**	**6.61±1.32**

Items rated on a scale from 1 = ‘‘much less” to 5 = ‘‘same” to 9 = ‘‘much more” attention needed compared to now.

*Statistically significant difference between male and female, P<0.05

There was no correlation between students’ perceptions for educational attention needs regarding ethically important practices and behaviors related to relationships and boundaries during the training (mean of the 12 items in Table 1) and the following: encountering ethical conflicts during training (r = 0.059, p = 0.547), having positive role models of ethical and professional behavior among supervising residents and faculty (r = -0.066, p = 0.497), being treated in an ethical and professional manner (r = -0.017, p = 0.860), overall role of medical education in helping to deal with ethical conflicts (r = 0.060, p = 0.536), and the extent of ethics training received at medical school (r = -0.026, p = 0.792).

Additional analyses dividing the respondents who scored a mean (mean of the 12 items in Table 1) of ≤5 (group 1, n = 26) and those with mean score >6 (group 2, n = 82). This revealed no significant differences (p = 0.59, 0.52, 0.39, 0.55, and 0.66 respectively) concerning each of the following five items: “To what degree, have you encountered ethical conflicts during training or practice”, “How much training in ethics have you received to date during your medical school education” (n = 59, 49 respectively), “How much has your overall medical education helped you to deal with ethical conflicts” (n = 51, 57 respectively), “During medical training, how many of your supervising residents and faculty have been positive role models of ethical and professional behavior” (n = 37, 71 respectively), and item “During medical training, how often have you been treated in an ethical and professional manner by your supervising residents, faculty, and training institution” (n = 34, 74 respectively).

### Reported needs concerning additional training on important topics pertaining to maintaining ethical relationships and boundaries during practices

Final year students indicated the need for more attention to all relationship and boundary topics during clinical practice (mean = 6.66±1.27). The topics with the most additional attention requested were “coping with mistakes in clinical care (mean = 7.25±1.63), reporting of medical mistakes (7.58±1.36), and balancing one’s personal and professional life” (mean = 7.05±1.74) (Table 2).

**Table 2:** Reported needs concerning additional training on ethically important topics pertaining to relationships and boundaries during practice (Mean±SD).

	Gender		Overall (N = 108)
Practices (α0.915)	Female (N = 84)	Male(N = 24)	Male and female
1. Accepting gifts from patients	6.10±2.08*	5.00±2.34	5.85±2.18
2. Sexual contact between patients and physicians	6.17±2.45	5.33±2.58	5.98±2.50
3. Drug companies supplying gifts and lunches	6.25±2.35	5.21±2.41	6.02±2.40
5. Interacting with patients’ families	6.80±1.60*	5.88±1.75	6.59±1.68
6. Confidentiality of medical records	6.87±1.80*	6.00±1.72	6.68±1.81
7. Responding to an impaired (e.g. drug or alcohol abuser) colleague	7.31±1.46*	5.88±1.51	6.99±1.58
8. Being asked to falsify clinical information	6.84±1.76	6.08±1.77	6.67±1.78
9. Resolving conflicts between allied health professionals	7.17±1.46*	5.75±1.51	6.85±1.58
10. Giving medical advice to friends & family	6.95±1.56*	6.04±1.57	6.75±1.60
11. Sexual harassment	6.95±1.71*	5.58±2.39	6.64±1.96
12. Personal relationships with patients	6.83±1.70*	4.58±2.24	6.33±2.06
13. Physician’s social & political responsibilities	7.04±1.75*	5.13±2.22	6.62±2.01
14. Coping with mistakes in clinical care	7.44±1.64*	6.58±1.44	7.25±1.63
15. Reporting of medical mistakes	7.71±1.37	7.13±1.26	7.58±1.36
16. Gender bias in clinical care	7.10±1.85*	5.54±2.47	6.75±2.10
17. Balancing one’s personal and professional life	7.30±1.63*	6.13±1.87	7.05±1.74
18. Writing prescriptions for friends, coworkers, or family members	6.67±1.98	6.00±2.09	6.53±2.01
19. Medicine as a profession (as opposed to other forms of work)	7.07±1.73*	5.87±2.36	6.81±1.94
**Group means**	6.93±1.20*	**5.74±1.09**	**6.66±1.27**

Items rated on a scale from 1 = ‘‘much less” to 5 = ‘‘same” to 9 = ‘‘much more” attention needed compared to now.

*Statistically significant difference between male and female, P<0.05

Women expressed a greater need for attention to all items compared with men (mean = 6.93±1.20 versus 5.74±1.09 female and male respectively) (Table 2). Statistically significant differences between the genders were mostly observed with women requesting more attention to “responding to an impaired (e.g. drug or alcohol abusing colleague) (p = 0.000), a physician’s social and political responsibilities” (p = 0.001).

There was no correlation between the ratings concerning the need for any additional educational attention during practice requested by students and the following: “encountering ethical conflicts during training (r = 0.096, p = 0.325), having positive role models of ethical and professional behavior among supervising residents and faculty (r = 0.008, p = 0.931), being treated in an ethical and professional manner (r = 0.073, p = 0.452), overall role of medical education in helping to deal with ethical conflicts’ (r = 0.003, p = 0.975), or for ‘the amount of ethics training received in medical school’ (r = - 0.056, p = 0.568).

Additional analyses showed no significant differences in the ratings between respondents who scored a mean of ≤5 (group 1) and those with mean score >6 (group 2) for the items “to what extent, have you encountered ethical conflicts during training (n = 26 and 82 respectively), How much training in ethics have you received to date during your medical school education (n = 59, 49 respectively), How much has your overall medical education helped you to deal with ethical conflicts (n = 51, 57 respectively), During medical training (n = 37, 71 respectively), how many of your supervising residents and faculty have been positive role models of ethical and professional behavior”, and the item “During medical training, how often have you been treated in an ethical and professional manner by your supervising residents, faculty, and training institution (n = 34, 74 respectively)” (p = 0.28, 0.28, 0.61, 0.88, 0.40 respectively).

## Discussion

This study assessed final year medical students’ perceived need for education regarding professional boundaries. Consistent with Lapid et al [5], who reported a perceived need for more education for psychiatric residents for the majority of topics pertaining to boundaries and relationships, our respondents also expressed interest in additional teaching for all the topics. In our study students strongly endorsed the need for a greater understanding of conflict resolution between attending physician and students, and dealing with mistreatment of medical students, interns and residents. With regard to the item on “reporting of medical mistakes”, respondents of both genders consistently ranked it as having the greatest need for further attention. This may reflect student’ concerns as to how they should behave in such circumstances that maybe encountered in clinical practice, particularly in interventional practices such as surgical procedures. Gap in the medical mistakes disclosure have been observed and several authors recommend disclosure of medical errors to be included in the teaching of medical ethics and professionalism. [31, 32] Students less frequently requested attention to topics involving sexually related issues and the reason is not clear but it may partly reflect a more settled opinion in the minds of the respondents. This also may rather follow some social or cultural values that limit open discussion about sexually related issues.

Consistent with findings from prior surveys of medical students, [10, 33] female medical students more strongly endorsed an interest in additional ethics education. The cause of these disparities is not clear, although it could be viewed as gender role or socialization differences between the genders. [10, 33] In a study by Feather [34] regarding the choice of Medicine as a career; working with people and improving society was more often the reason for women, while high social and economic status was the reason for men. Furthermore, Bickel and Ruffin [35] reported that women are more likely declare inadequate curriculum coverage for many topics compared to male counterparts.

Our data showed no correlation between the overall role of current medical education in helping to deal with ethical conflicts, nor for the extent of ethics training received in medical school and the greater attention to the subject requested by students. This should be interpreted with caution as this could be attributed to the fact that all respondents in our study had similar curricular exposure and assessing the effect of the true extent of training would have been difficult. This also could, perhaps, be explained by the fact that our students have not yet been so deeply involved in patient care, and are not fully aware of ethical dilemmas; this may be more evident as they advance in their training.

A potential limitation of the study is that students’ views were explored once by each cohort 2009 to 2013 at a single medical school, implying thoughtful generalizations of the findings is warranted. Based on a growing body of literature [36] regarding unprofessional and unethical behaviors among practicing physicians and medical students including boundary transgressions [37, 38] together with some knowledge of other medical school curricula in the region and beyond we nevertheless believe the findings of this study are representative of curricular needs at other medical schools. Medical students more in general are in need of explicit support and guidance on how to deal with these issues. Another limitation was that the potential for entry to medical school is similar for male and female, however, fewer male applications are received and admitted each year. Our study included 24 males and 84 females. Hence, generalization of these findings should be with caution.

The study’s strength is that it supports the view that assessing trainees’ perspectives and preferences is important for curricular improvement. [5, 10, 11, 39] Although medical schools are not expected to teach all topics in the limited duration of six years, there is an obligation to equip graduates with basic skills to solve ethical issues at a time when professional boundaries are gaining greater professional and public attention. Indeed, the literature indicates that exposure to unethical and unprofessional behavior is thought to play a significant role in cynicism and unprofessional behaviors of medical students in training. [40] Future research focus is therefore recommended on contributory barriers and enabling factors in medical school curricula generally and among teaching faculty specifically for exploitation of opportunities to role model and teach the topics of professional ethics, professional boundaries and unprofessional behaviors more systematically. Additionally, further research beyond the educational context is warranted. Since, the literature on unethical behaviors suggest dispositional and contextual factors such as character, personality, environmental culture, etc. may influence individual’s propensity to act unethically and unprofessionally in the workplace [37, 38, 40–44] we recommend future research be conducted in healthcare environments where relationship boundaries and professionalism transgressions may be observed.

## Conclusion

Medical students recognized the need for more education and training in the undergraduate medical ethics curriculum regarding relationship boundaries. Being aware of students’ perspectives is essential as it may contribute to curricular improvement, which may in turn assist them to fulfil their professional roles during the care of patients.

## Supporting information

S1 Checklist STROBE 2007 (v4) statement—checklist of items that should be included in reports of *cross-sectional studies*. (DOCX)

S2 Checklist (PDF)
