# Supplementary material for: Relationships and boundaries: Learning needs and preferences in clerkship medical environments
Source: PLoS One. 2020 Jul 20;15(7):e0236145. doi: 10.1371/journal.pone.0236145 (PMC7371200; doi:10.1371/journal.pone.0236145)
Supplement: S2 Checklist — (PDF) [file pone.0236145.s002.pdf]

There are techniques to increase response rates. A questionnaire must be concise and easy to understand, reminders should be sent out, and method of recruitment should be carefully considered. Sitzia and Wood [13] found that participants recruited by mail or who had to respond by mail had a lower mean response rate (67%) than participants who were recruited personally (mean response 76.7%). A most useful review of methods to maximize response rates in postal surveys has recently been published [14].

Data collection

Researchers should approach data collection in a rigorous and ethical manner. The following information must be clearly recorded:

- How, where, how many times, and by whom potential respondents were contacted.
- How many people were approached and how many of those agreed to participate.
- How did those who agreed to participate differ from those who refused with regard to characteristics of interest in the study, for example how were they identified, where were they approached, and what was their gender, age, and features of their illness or health care.
- How was the survey administered (e.g. telephone interview).
- What was the response rate (i.e. the number of usable data sets as a proportion of the number of people approached).

Data analysis

The purpose of all analyses is to summarize data so that it is easily understood and provides the answers to our original questions: 'In order to do this researchers must carefully examine their data; they should become friends with their data' [15]. Researchers must prepare to spend substantial time on the data analysis phase of a survey (and this should be built into the project plan). When analysis is rushed, often important aspects of the data are missed and sometimes the wrong analyses are conducted, leading to both inaccurate results and misleading conclusions [16]. However, and this point cannot be stressed strongly enough, researchers must not engage in data dredging, a practice that can arise especially in studies in which large numbers of dependent variables can be related to large numbers of independent variables (outcomes). When large numbers of possible associations in a dataset are reviewed at $P < 0.05$, one in 20 of the associations by chance will appear 'statistically significant'; in datasets where only a few real associations exist, testing at this significance level will result in the large majority of findings still being false positives [17].

The method of data analysis will depend on the design of the survey and should have been carefully considered in the planning stages of the survey. Data collected by qualitative methods should be analysed using established methods such as content analysis [18], and where quantitative methods have been used appropriate statistical tests can be applied. Describing methods of analysis here would be unproductive as a multitude of introductory textbooks and on-line resources are available to help with simple analyses of data (e.g. [19, 20]). For advanced analysis a statistician should be consulted.

Reporting

When reporting survey research, it is essential that a number of key points are covered (though the length and depth of reporting will be dependent upon journal style). These key points are presented as a 'checklist' below:

(1) Explain the purpose or aim of the research, with the explicit identification of the research question. YES
(2) Explain why the research was necessary and place the study in context, drawing upon previous work in relevant fields (the literature review). YES
(3) Describe in (proportionate) detail how the research was done.
  (a) State the chosen research method or methods, and justify why this method was chosen. YES
  (b) Describe the research tool. If an existing tool is used, briefly state its psychometric properties and provide references to the original development work. If a new tool is used, you should include an entire section describing the steps undertaken to develop and test the tool, including results of psychometric testing. YES
  (c) Describe how the sample was selected and how data were collected, including:
    (i) How were potential subjects identified? YES
    (ii) How many and what type of attempts were made to contact subjects? YES
    (iii) Who approached potential subjects? YES
    (iv) Where were potential subjects approached? YES
    (v) How was informed consent obtained? YES
    (vi) How many agreed to participate? YES
    (vii) How did those who agreed differ from those who did not agree? YES
    (viii) What was the response rate? YES
(4) Describe and justify the methods and tests used for data analysis. YES
(5) Present the results of the research. The results section should be clear, factual, and concise. YES
(6) Interpret and discuss the findings. This 'discussion' section should not simply reiterate results; it should provide the author's critical reflection upon both the results and the processes of data collection. The discussion should assess how well the study met the research question, should describe the problems encountered in the research, and should honestly judge the limitations of the work. YES
(7) Present conclusions and recommendations. YES

The researcher needs to tailor the research report to meet:
